# IL-17A and its homologs IL-25/IL-17E recruit the c-RAF/S6 kinase pathway and the generation of pro-oncogenic LMW-E in breast cancer cells

**DOI:** 10.1038/srep11874

**Published:** 2015-07-08

**Authors:** Sarah Mombelli, Stéphanie Cochaud, Yacine Merrouche, Christian Garbar, Frank Antonicelli, Emilie Laprevotte, Gilles Alberici, Nathalie Bonnefoy, Jean-François Eliaou, Jérémy Bastid, Armand Bensussan, Jérôme Giustiniani

**Affiliations:** 1 Institut National de la Santé et de la Recherche Médicale (INSERM) U976, Hôpital Saint Louis, 75010 Paris, France; 2 Université Paris Diderot, Sorbonne Paris Cité, Laboratoire Immunologie Dermatologie & Oncologie, UMR-S 976, F-75475, Paris, France; 3 Institut Jean Godinot, Unicancer, F- 51726 Reims, France; 4 Université Reims-Champagne-Ardenne, DERM-I-C, EA7319, 51 rue Cognacq-Jay, 51095 Reims Cedex, France; 5 OREGA Biotech, F-69130 Ecully, France; 6 IRCM, Institut de Recherche en Cancérologie de Montpellier; INSERM, U896; Université Montpellier 1; CRLC Val d’Aurelle Paul Lamarque, Montpellier, F-34298, France; 7 Département d’Immunologie, Centre Hospitalier Régional Universitaire de Montpellier et Faculté́ de Médecine Université Montpellier 1, Montpellier, France

## Abstract

Pro-inflammatory IL-17 cytokines were initially described for their pathogenic role in chronic inflammatory diseases and subsequent accumulating evidence indicated their involvement in carcinogenesis. In the present study we report that IL-17A and IL-17E receptors subunits mRNA expressions are upregulated in breast cancers versus normal samples. IL-17E, which is undetectable in most normal breast tissues tested, seems more expressed in some tumors. Investigation of the molecular signaling following stimulation of human breast cancer cell lines with IL-17A and IL-17E showed that both cytokines induced the phosphorylation of c-RAF, ERK1/2 and p70 S6 Kinase were involved in the proliferation and survival of tumor cells. Accordingly, IL-17A and IL-17E promoted resistance to Docetaxel and failed to induce apoptosis as previously reported for IL-17E. Interestingly, we also revealed that both cytokines induced the generation of tumorogenic low molecular weight forms of cyclin E (LMW-E), which high levels correlated strongly with a poor survival in breast cancer patients. These results show for the first time some of the molecular pathways activated by IL-17A and IL-17E that may participate to their pro-oncogenic activity in breast cancers.

The IL-17 cytokine family is composed of six members, IL-17A to IL-17F with IL-17A as the prototypic one^1^. A total of five receptors have been described, IL-17RA to IL-17RE. IL-17A binds and signals through the IL-17RA/IL-17RC receptor heterodimer, whereas IL-17E, also named IL-25, is a ligand for the IL-17RA/RB heterodimer^2^. IL-17A is mainly produced by T helper 17 (TH17) cell subset and by innate immunity lymphocytes including TCR-γδ^+^ T cell, iNKT, lymphoid tissue inducer (LTi) cells, CD3^−^NKp46^+^ lymphocytes or neutrophils that are potentially responsible for initiating pathogenic TH17 cells proliferation^1^,^3^,^4^,^5^. A growing body of evidence indicated important roles for this cytokine and TH17 cells in the development of allergic and autoimmune diseases as well as in protective mechanisms against bacterial and fungal infections^6^ and gained prominence in cancer, particularly in breast carcinomas^7^,^8^,^9^. Mouse models of breast cancers revealed that IL-17A promotes tumor growth and angiogenesis^10^,^11^. Recently, we have shown that IL-17A produced by tumor infiltrating lymphocytes promotes breast cancer cell chemoresistance and proliferation through activation of ERK1/2 pathway^12^,^13^ . Interestingly, it has been reported that IL-17B produced by malignant cells could also promote cancer cell survival through activation of NF-κB^14^,^15^. In contrast IL-17E was reported to be produced by normal mammary epithelial cells, and its binding to IL-17RA-IL-17RB complex induced breast cancer cell apoptosis^15^. Thus, it was suggested that IL-17E production by normal epithelium might prevent the emergence of transformed epithelial cells by inducing malignant cell apoptosis, while IL-17B produced by transformed cells promoted cancer cell survival by displacing IL-17E from its receptor.

In the present study, we aimed to identify in breast cancer cells the signaling pathways recruited following IL-17A and IL-17E cytokine stimulation. The results revealed that both cytokines activated similar oncogenic pathways in breast malignant cell lines leading to Docetaxel resistance and generation of LMW cyclin E. In contrast to previous report, we failed to found IL-17E expression by non-transformed epithelial cells and to reproduce its potential induction of breast cancer cell apoptosis. These results shed new light on the potential role of IL-17A and IL-17E in breast cancer and further studies should contribute to understand whether they could be potential therapeutic targets. Furthermore, these data question the role of IL-17E as a potential tumor suppressor.

## Results

### Expression of IL-17E and its receptor in breast cancer biopsies and cell lines

**Figure 1:**
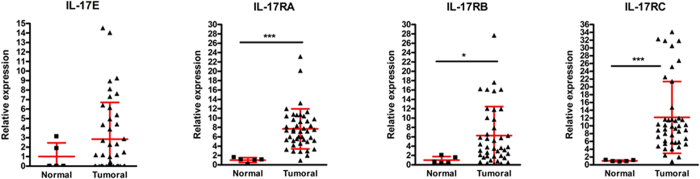
Expression of IL-17 cytokines and receptors in clinical samples. The TissueScan Breast Tissue qPCR array was used to determine transcript levels of the IL-17 cytokines (IL-17A and IL-17E) and their receptors (IL-17 RA, IL-17 RB and IL-17 RC). The breast tissue scan contains 48 tissues covering 4 diseases stages and normal tissues. The target transcript levels were normalized to β-Actin and calibrated to the mean mRNA level (arbitrary value of 1) in normal tissue. Data were compared using student’s *t* test (*P < 0.05, **P < 0.01, ***P < 0.001).

To elucidate the potential role of IL-17E in breast cancer, we first assessed the expression of this cytokine and the IL17-RA, RB and RC receptor subunits in human normal and cancer breast tissues, using RT-QPCR. As illustrated in Fig. 1 IL-17E mRNA, which is undetectable in most normal breast tissues tested, seems more expressed in some tumors. Furthermore, the three IL-17R subunits, corresponding to the IL-17E (IL17RA/RB) and IL-17A (IL17 RA/RC) receptors, were highly upregulated in tumor versus normal samples, suggesting that IL-17E as IL-17A signaling is potentially active in human breast cancer.

**Figure 2:**
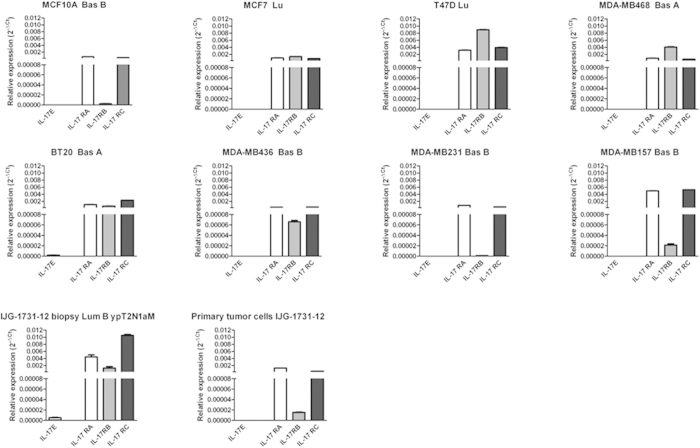
Expression of IL-17 cytokines and receptors in human breast cell lines. Real-Time RT-PCR analysis of the IL-17 cytokines mRNA (IL-17A and IL-17E) and their receptors (IL-17 RA, IL-17 RB and IL-17 RC) in different human beast cell lines. Expression was normalized to the GAPDH mRNA expression. Data are the mean +/− SEM of one experiment performed in duplicate.

We then asked whether the cytokine and IL-17 receptor subunits are expressed by the tumor cells. To address this question, we assessed the expression of IL-17E, IL-17RA, IL-17RC and IL-17RB in various human breast cancer cell lines as well as in non-transformed mammary epithelial cells MCF10A, in primary tumor cells (IJG-1731) derived *ex vivo* from an ER-negative breast cancer biopsy. As illustrated in Fig. 2, the expression of the IL-17RA and IL-17RC receptor subunits was ubiquitous as all the primary cells and cell lines tested expressed high levels of IL-17RA and IL-17RC. In line with the results obtained from biopsies (Fig. 1), IL-17RB was undetectable in non-transformed MCF10A cells and significantly upregulated in most breast cancer cell lines tested; suggesting that increased expression of IL17RB could be a malignant trait.

Further, we found that cell lines and the primary tumor cells did not expressed IL-17E transcripts, indicating that the cytokines are likely to be produced by the tumor microenvironment. Although a previous report^15^ indicated that non-transformed MCF10A mammary epithelial cells expressed IL-17E, we were unable to detect IL-17E transcript in this cell line (Fig. 2).

### IL-17E fails to induce breast cancer cell apoptosis and promoted chemoresistance

Furuta *et al.* reported that non-transformed mammary epithelial cells express IL-17E and low levels of IL-17RB, and this cytokine induced apoptosis in transformed breast cells (with upregulated IL-17RB) but not in non-transformed cells, thereby serving as a tumor suppressor. Although we here confirmed that IL-17RB expression is much higher in tumor cells than in non-transformed cells, we were unable to detect IL-17E mRNA in MCF10A cells (Fig. 2). IL-17E was even express at higher levels in tumor biopsy specimens than in normal breast tissues (Fig. 1). These results raised questions about the potential role of IL-17E as a tumor suppressor.

**Figure 3:**
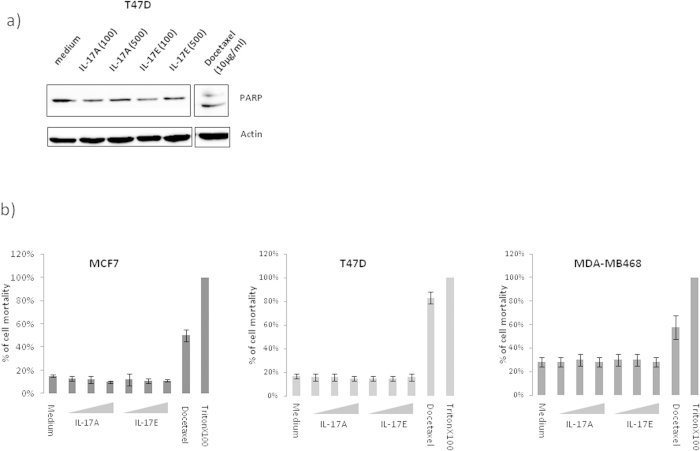
IL-17E do not induce apoptosis. Cell apoptosis was analyzed by detection of PARP proteolysis (**a**) or measurement of LDH released into cell supernatants (**b**) to this aim; cells were treated 24 h in a serum free medium with IL-17A or IL17-E at 20, 100 or 500 ng/ml. As positive control for PARP cleavage or LDH detection we used Docetaxel at 10 μg/ml.

We therefore decided to test the ability of IL-17E to induce apoptosis of breast cancer cell lines, and used IL-17A as negative control as we previously demonstrated that it fails to induce apoptosis by itself^12^. As illustrated in Fig. 3A, we analyzed cell apoptosis by evaluating PARP proteolysis in the representative T47D malignant cell line treated with IL-17E or IL-17A either at 100 ng/ml or 500 ng/ml. Treatment with Docetaxel at 10 μg/ml was used as a positive control for apoptosis. The results clearly show that, in contrast to Docetaxel, neither IL-17A nor IL-17E induced PARP cleavage at the indicated concentrations.

Next, to further confirm the lack of apoptosis induced by IL-17A and IL-17E, we measured the level of cell death of MCF7, T47D and MDA-MB468 cells cultured in presence of recombinant IL-17A or IL-17E used at concentrations from 20 ng to 500 ng/ml. Docetaxel at 10 μg/ml and 1% Triton × 100 were used as positive controls. Results presented in Fig. 3B revealed that IL17A and IL-17E did not induce the cell death of MCF-7, T47D or MDA-MB468 cells.

**Figure 4:**
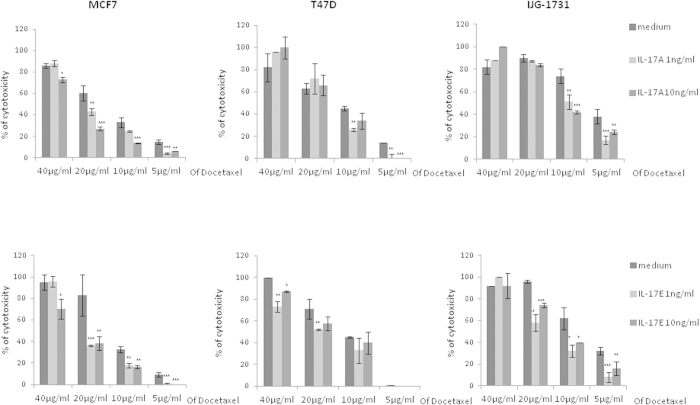
IL-17E induces cell chemoresistance to Docetaxel. Breast cancer cells were cultured in complete medium alone (medium) or supplemented with IL-17A or -E for 48 h. Cells were then switched in FCS-free medium supplemented with the respective cytokine for 24 h before adding Docetaxel at various concentration for 7 h at 37 °C. The cytotoxicity was determined using the Cytotoxicity Detection Kit (Roche). Data shown are representative of 3 independent experiments. (*P < 0.05; **P < 0.01; ***P < 0.001).

Although IL-17E did not induce apoptosis of IL-17RB expressing breast cancer cells, we tested whether it could modulate cell death induced by chemotherapy drugs. As we previously reported that 1 to 10 ng/ml of IL-17A was very potent at inhibiting Docetaxel induced cell death in various human breast cancer cell lines^12^.The results shown in Fig. 4 indicated that pretreatment of MCF-7, T47D or primary tumor cells IJG-1731 with 1 to 10 ng/ml of IL-17E consistently results in decreased Docetaxel-induced cell death.

### IL-17A and IL-17E promotes the phosphorylation of c-Raf, ERK and p70S6 kinase

**Figure 5:**
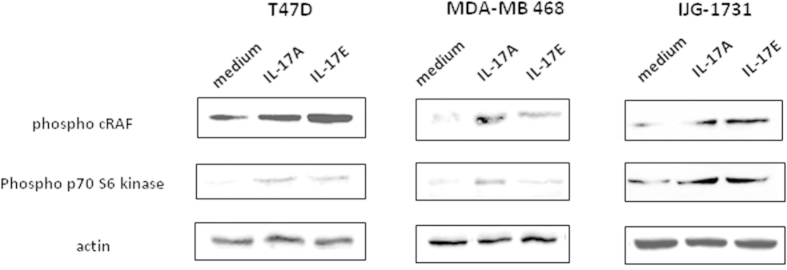
IL-17A and IL-17E activate c-Raf and p70S6 kinase. Activation of c-Raf (pSer338) and p70S6 kinase (pThr 389) was analyzed successively by Western Blot (WB) on T47D, MDA-MB468 and IJG-1731 cell lines. Cells were treated with either IL-17A or IL-17E at 20 ng/ml for 20 min before cell lysis. 70 μg of protein were loaded for each condition and loading control was done with an anti β-actin mAb. Data are representative of 3 independent experiments and all the lines were run on the same gel and under the same experimental procedure.

In order to identify the kinases involved in chemoresistance induced by IL-17A and IL-17E pre-treatments, we explored the activation of signaling pathways involved in cancer progression. To this aim, MCF-7, T47D, and primary tumor cells IJG-1731 cells were starved overnight then treated with the cytokines. We analyzed their phosphorylation status for 20 min following the cytokine treatment. In cell lines tested, we observed an activation of the MAP kinases pathway through the phosphorylation of c-RAF on Serine 338 (p-cRAF) (Fig. 5) and p42/p44MAPK (suppl Figure 2A and 2B)^12^. In untreated IJG-1731 and MDA-MB468 cells p-cRAF was almost undetectable and was highly induced after addition of cytokines. 120% up to 1000% increased signal was obtained, depending on the IL-17 cytokine used.

It should be mentioned that c-RAF serine 338 is phosphorylated by the p21 activated kinase^16^ and corresponded to similar phosphorylated sites in A-Raf (Ser299) and B-Raf (Ser445)^17^.

Because the PI3K/mTOR/p70S6K axis is well known to play an important role in cell growth and survival, and is described to be involved in breast cancer cell proliferation and chemoresistance^18^, we looked at the activation of the p70S6K (p70^S6K^), which regulates cell cycle, inhibits the pro-apoptotic protein BAD and controls protein synthesis through the phosphorylation of the S6 protein of the 40S ribosomal subunit^19^. We measured the phosphorylation of Thr389, which closely correlates with p70 kinase activity *in vivo*^20^. The results showed in Fig. 5 indicated that incubation with IL-17A or Il-17E enhanced the phosphorylation of on Thr389 p70^S6K^, in T47D, MDA-MB 468 and IJG-1731 cell lines (Fig. 5). We could not reproduce this result with MCF7 cell line due to the constitutive phosphorylation of the S6 kinase (data not shown).

As p70S6K may also be activated by the c-RAF/ERK pathway, we chemically blocked p42/p44MAPK with the U0126 MEK specific inhibitor to further demonstrate the contribution of the c-RAF, ERK, p70S6K pathway in IL-17-induced chemoresistance in breast cancer cell lines. The results in Supp Figure 1 show that lack of ERK1/2 activation in the BT20 breast cancer cell totally abolished the chemoresistant effect induced by IL-17E cell stimulation.

### IL-17A or IL-17E treatment enhances the generation of low molecular weight forms of cyclin E (LMW-E) in breast tumor cell line

As we found that breast malignant cell proliferation was increased by IL-17A or IL-17E (suppl Fig. 2C), we next investigated the expression of the full length cyclin E, a regulatory subunit of Cdk2 that contributes to G_1_/S transition, as well as the expression of LMW forms of cyclin E for which the regulatory domain is missing^21^,^22^.

**Figure 6:**
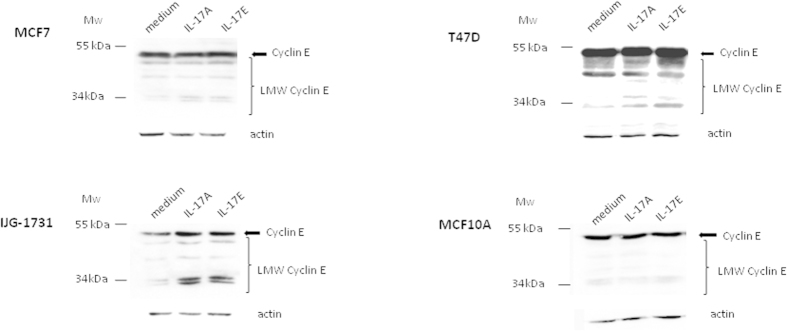
IL-17A or IL-17E enhance the generation of low molecular weight forms of cyclin E (LMW-E). LMW-E generation was analyzed by WB on MCF-7, T47D, IJG-1731 and MCF10A after 72 h of treatment with Il-17A or IL-17E at 20 ng/ml. 70 μg of protein were loaded for each condition and loading control was done with an anti β-actin mAb after stripping. Almost all of the five published short forms ranging from 34 to 49 kDa, were induced. No induction was found for the nonmalignant MCF10A cell line suggesting a specific feature of tumor cells. Data are representative of 3 independent experiments and all the lines were run on the same gel and under the same experimental procedure.

Interestingly, we found that IL-17A or IL-17E did not increase the expression of the full length cyclin E in MCF7, T47D and MCF10A cell lines, when compared to untreated cells. In contrast, we observed a consistent increase of cyclin E in the primary breast cancer cell line IJG-1731 when cultured with either IL-17A or IL-17E (Fig. 6). More importantly, we found that all breast cancer cell lines upregulated the generation of LMW-E forms. Although some of LMW-cyclin E forms could be weakly present at basal level, we detected almost all of the five published short forms^23^, ranging from 34 to 49 kDa, after 72 h of treatment with each cytokines. Cyclin E proteolysis might be a tumor specific mechanism as IL-17A failed to up-regulate the generation of LMW-E forms in the non-transformed mammary epithelial MCF10A cells although these cells express significant levels of IL-17RA and IL-17RC (Fig. 1). Of note, MCF10A cells lack IL-17RB and did not respond to IL-17E.

## Discussion

Interleukins are known to promote tumor growth and metastasis, and IL-17A could be a typical example. This cytokine produced by infiltrating lymphocytes has been shown to promote tumor proliferation^10^,^12^ as well as migration, invasion and resistance to chemotherapy^12^ and to anti-angiogenic treatment^24^. Recent works focused on IL-17B and IL-17E, which are found in breast tumor microenvironment, have reported opposite functions. Although they share the IL-17RB chain as co receptor, pro-oncogenic features were described for the breast tumor cell secreted IL-17B, whereas tumor suppressor role was associated to the normal breast epithelial cells secreted IL-17E. Here, we investigated the action of IL-17A and IL-17E on breast cancer cell lines and cell signaling events induced after recruitment of IL-17RA/IL-17RC or IL-17RA/IL-17RB receptors.

We found that IL-17E receptor mRNA was increased in malignant breast tissues and breast cell lines as compared to healthy tissues and that IL-17E mRNA seems expressed in some of tumors. Neither IL-17E nor IL-17A stimulation induced cell apoptosis. The absence of apoptosis was not due to the inability of the cytokine to signal through the IL-17E receptor as the E-coli-produced IL-17E, similarly to IL-17A^12^, activated the ERK pathway in breast cancer cells (supp Figs 2A and 2B). Moreover, a potential cross reactivity between IL17A receptor (IL17RA/RC) and IL-17E is unlikely, since this cytokine, on the contrary to IL-17A, is not able to induce any signaling event on IL17RB^neg^ MDA-MB231 cells (data not shown). These findings are contradictory to published data^15^,^25^ showing a pro-apoptotic effect of E-coli-derived IL-17E on breast cancer cell line expressing IL-17RB such as MDA-MB468. A potential explanation for these discrepancies is that all our assays were performed with physiological compatible IL-17E concentrations (1–10 ng/ml), while *In vitro* pro-apoptotic effects were observed with higher concentrations (250 up to 2000 ng/ml) in the study of Vahid Younesi *et al.*^25^.

In our study, not only IL-17E did not display a pro-apoptotic role, but we also demonstrated that IL-17E pretreatment was capable to partially prevent drug sensitivity in association with the activation of the p70S6K, ERK and c-RAF pathway. Activation of b-RAF have been already described in tumor cells^26^ and it is interesting to underline that c-RAF and b-RAF are interconnected and their heterodimerization increases the activity of both kinases^27^,^28^. Our results are in agreement with Huang *et al.* indicating that IL-17RB has an important role in breast tumorigenesis and that its high expression correlates with shorter survival. Of note, such activation was also observed when cells were pretreated with IL-17A. Furthermore, the chemoresistant phenomenon observed was not drug specific as IL-17E also protected breast cancer from doxorubicin-induced toxicity (data not shown). Thus, our results support the hypothesis that both IL-17A and IL-17E are involved in breast cancer progression by activating common signaling cascade end point despite the fact these cytokines trigger distinct receptors.

Conversely to previous studies^15^,^25^, we demonstrated that IL-17E displayed a protumoral role in breast cancer. Such discrepancies in the function of a single molecule are quite puzzling, and might rely on its environment. In this regard, it is worth to note that IL-17 was not secreted by tumoral but by infiltrating cells in breast human biopsy. Conversely, Furuta *et al.* demonstrated a potent anticancer activity of purified IL-17E in mouse cancer models^15^. Interestingly, previous work already demonstrated that IL-17E exhibits potent antitumor effects against various human cancer cell lines (including the MDA-MB-435 breast cancer cells) *in vivo*^29^. Although the mechanisms were not fully elucidated, antitumor activity was evidenced in nude mice (normal B cells) but lost in SCID mice (no B cells), suggesting that the anti-tumoral effects of IL-17E were B-cell dependent. Tumor infiltrating B cells were extensively studied in breast cancer, where they are present in ~25% of tumors and could represent up to 40% of the TIL population^30^. Moreover in node negative breast cancer, presence of TIL-B was positively associated with survival^31^. The recruitment of IL-17RB, expressed on B cells^32^, could participate to the homing of such cells in the tumor microenvironment and positively regulates the response observed in nude mice. An IL-17E enhanced cytotoxicity of TIL-B cells could be discussed as well as it was already demonstrated that B cells stimulated with IL-21 can secrete granzyme B and could have a direct cytotoxicity against tumor cells^33^. Therefore, in breast cancer IL-17E could display antagonistic effects on tumour progression, a direct pro-tumoral effect on breast cancer cells and an indirect anti-tumoral effect via B-cell recruitment and activation.

Noteworthy, in our study the IL-17E protumoral effects were characterized by an enhanced proliferation capacity of breast cancer cells. During the last decade, it was shown that high expression of low molecular form of cyclin E (LMW-E) corresponded to a poor disease free survival factor in breast cancers^26^. LMW-E forms, generated by proteolysis from the full length cyclin E, highly enhance CDK2 activity and are not inhibited by p21 and p27 proteins^22^. Here, we report for the first time that both IL-17E and IL-17A triggered signaling cascades converging towards the generation of LMW-E forms. Such stimulation was only observed with tumor cell lines, opening the possibility that anti-IL-17 therapy could specifically target cancer cells. These active forms of cyclin-E are highly oncogenic as demonstrated by Duong. T *et al.*, in mice model, where ectopic LMW-E expression on hMEC cell line renders it tumorogenic and induces the development of mammary carcinoma and metastasis, whereas the full length expression of cyclin E do not^26^. Two enzymes have been involved in the LMW-E generation; calpain and an elastase like protease which is inhibited by endogenic Elafin a critical component of the epithelial barrier against neutrophil elastase^23^,^34^. We could thus hypothesize that IL-17A/E-induced signaling cascades could affect the protease/anti-protease balance both by up regulating the elastase like protease expression and down regulating Elafin expression in breast cancer cells compared to normal cells^35^. Such tumor specific pro-oncogenic effects could involve direct and indirect mechanisms. LMW-E could both originate from the c-RAF/ERK pathway activation triggered by IL-17, but also from other signaling cascades that control the protease/antiprotease balance in the tumour microenvironment. Whether these latter regulations are direct or indirect through the release of other molecules that could interfere by autocrine/paracrine mechanisms still need to be determined. In this setting, our data suggest that several IL-17 family members, present in the tumor breast cancer microenvironment, may both act as survival factors and promote chemoresistance. It was reported that IL-17A and IL-17B neutralizing antibody treatments led to decreased breast cancer growth in mice. It would be interesting to test whether anti-IL-17A, IL-17B and/or IL-17E based therapies could affect tumor growth and sensitivity to chemotherapy and immunotherapy (anti-HER2) in animal models of cancer. To that matter, further work on the expression of IL-17 receptors from metastatic breast cancer cells compared to primary tumor could be informative, especially for liver and lung metastasis where IL-17E is expressed^36^,^37^.

Taking together the findings reported here suggest that IL-17A and IL-17E are expressed and active in breast cancer and may participate to tumorogenesis.

## Materials and Methods

### Cell lines and culture conditions

T47D (ATCC No. HTB-133) cells and MCF7 (ATCC No. HTB-22), BT-20 (ATCC No HTB-19) and IJG-1731 cells were cultured in a complete RPMI-1640 media with L-glutamine, supplemented with 10% fetal calf serum (FCS) and penicillin–streptomycin solution (100 μg/ml each) (Life technology,Saint-Aubain, France). All cells were cultivated in a humidified 5% CO2 incubator at 37 °C. Cells were grown for 4–5 days until confluency. Cells were harvested with 0.25% Trypsin–EDTA solution (Life technology, Saint-Aubain, France) and then passed into new T-75 tissue culture flasks. Starvation medium did not contain FCS.

### Generation of IJG-1731 cell line

IJG-1731 cell line was obtained from a LumB tumor characterized as a ypT2N1aM tumor. Briefly, biopsy was cut and trypsinized to liberate tumor cells. Cell preparation obtained was then cultured in complete RPMI1640 medium. After several weeks of cell culture stabilization, cells were phenotyped as negative for eostrogen, progesterone and HER2 receptors and positive for EGFR (HER1).

### Antibodies

Rabbit Anti-phospho Ser388 c-RAF (clone 56A6), mouse anti-phospho Thr389 P70 S6 kinase (clone 1A5), rabbit phospho Erk1/2 (Thr202/Tyr204) (D13.14.4E), rabbit Erk1/2 (137F5), mouse anti-Cyclin E (clone HE12), polyclonal rabbit anti-PARP/cPARP and rabbit anti-Actin (clone 13E5) were obtained from Cell Signaling Technology (Leiden, Netherlands).

### Proliferation assay (^3^H)

400 cells per well (total volume of 200 μl) were seeded in a 96 wells plate with medium alone or completed with cytokines at 20 ng/ml during 72 hours at 37 °C, 5% CO2 After 72 h of culture, cells were pulsed for 16 h with 1 μCi of tritiated thymidine ([3H]-TdR) per well. [3H]-TdR uptake was measured using MicroBeta2 plate counter (PerkinElmer, Courtaboeuf, France). All conditions were done in triplicate.

### Immunoblotting

#### c-RAF and S6 kinase

To detect phosphorylation of c-RAF, and p70S6 kinase 3.10^5^ cells per well 6 wells plate are seeded in 2 ml of complete medium 24 h then cultured overnight in starvation medium. Medium is removed and cells are activated with cytokine at 20 ng/ml (1 ml) in free FCS RPMI. After an incubation of 20 min at 37 °C, the cells were lysed in 1% Triton × 100 lysis buffer, incubated for 1h on ice and centrifuged at 4 °C for 10 min at 10,000 g. The supernatants were collected and protein concentration was determined using the Bradford Assay (Bio-Rad, Marnes la Coquette, France). Proteins (70 μg) were resolved in 8% SDS–PAGE and transferred on nitrocellulose membrane. The membrane was blocked for 1 h at room temperature, by using 5% nonfat milk in Tris-buffered saline (TBS) containing 0.1% Tween20 (Sigma–Aldrich) and incubated overnight at 4 °C with a anti-phospho-c-RAF antibody (1:1,000), anti-phospho S6 kinase (1:1,000) or with an anti-Actin antibody (1:1,000) (Cell Signaling, Leiden, Netherlands) used as a loading control, in blocking solution. After 3 washes, the membrane was incubated 1h at room temperature with goat anti-rabbit or goat anti-mouse secondary antibodies (1:10,000) (Jackson Immunoresearch, West Grove, USA) conjugated to horseradish peroxydase.

#### Cyclin E

To detect multiple forms of Cyclin E, cells were seeded in a 6 wells plate in 2 ml of starvation medium then cultured overnight. Cells are activated with cytokines diluted at 20 ng/ml in a 3% FCS medium for 72 hours. Cells are then treated as described above.

#### PARP/cleaved PARP

3.10^5^ cells per well 6 wells plate are seeded in 2 ml of complete medium 24 h then cultured overnight in starvation medium. Medium is removed and cells are activated with cytokine at 100 or 500 ng/ml (1 ml) in free FCS RPMI. A positive control was performed by treating cells with Docetaxel at 10 μg/ml. After 24 h of culture, the medium is removed and cells are immediately lysed as described previously.

For each sample, 70 μg of protein were subjected to 8% SDS-PAGE under reducing conditions. Resolved proteins were transferred onto nitrocellulose membrane which was blocked in 5% milk/TBS Tween20 0.1% (TBS-T) solution for 1 h at RT. Primary antibodies were added overnight at 4 °C in 5% Bovine Serum Albumin (BSA)/TBS-T solution and extensively washed with TBS-T. Corresponding horseradish peroxidase conjugated anti-mouse or anti-rabbit antibodies (Jackson Immuno Research) were used at dilution 1:10 000 for 1 h at RT before final wash with TBS-T and subsequent detection of protein bands using SuperSignal West Pico Chemoluminescent kit (Thermo Scientific, Rockford, USA). All experiments were performed in triplicates.

### cDNA synthesis

Total RNA was isolated using the GenElute™ Mammalian Total RNA kit (Sigma–Aldrich, Saint-Quentin Fallavier, France) following the manufacturer’s instructions. Total RNA (1 μg) was treated with 1 U/μg RNA of DNase I Amplification Grade (Life Technologies, Saint-Aubain, France) according to the manufacturer’s instructions, and in the presence of 10 U/μg RNA of RNaseOUT (Life Technologies, Saint-Aubain, France). After DNase inactivation, RNA was reverse transcribed using random nonamers (Sigma–Aldrich, Saint-Quentin Fallavier, France) and M-MLV Reverse Transcriptase H Minus (Promega, Charbonnières, France) according to the manufacturer’s instruction.

### Polymerase chain reaction of reverse transcribed mRNA

The forward and reverse primers used in the PCR reaction were designed with Primer-BLAST software (http://www.ncbi.nlm.nih.gov/tools/primer-blast/), except for IL-17A^38^ and GAPDH^39^ and were listed in Table 1.

Real-time polymerase chain reaction quantification was carried out with the LightCycler 480 II System (Roche Diagnostics, Meylan, France) using the SYBR Premix Ex Taq (TliRNaseH Plus) kit (Ozyme, Saint Quentin, France). The cycling conditions were as follows: 95 °C for 1 min followed by 40 cycles of 95 °C for 20 s, 60 °C for 20 s and 72 °C for 30 s. The sizes of the RT-PCR products were confirmed by agarose electrophoresis. At the end of the amplification, a melting temperature analysis of the amplified gene products was performed routinely for all cases; the PCR products were melted by gradually increasing the temperature from 60 to 95 °C in 0.3 °C steps, and the dissociation curves were generated with the Melting Curve analysis tool of the LightCycler 480 software (Roche Diagnostics, Meylan, France). We confirmed that only one product was consistently amplified in all PCR reactions. The negative water control showed no amplification. The relative expression of the genes of interest normalized to GAPDH was determined by the delta Ct method.

### Tissue qPCR array

The target genes expression was also analyzed in normal and tumoral breast tissues using the TissueScan Breast Tissue qPCR array (BCRT502, OriGene Technologies, Rockville, USA). This tissue scan is a panel of normalized cDNA from 5-normal and 42 different stages of breast cancer tissues. A description in depth pathology report (including histology sections) for all of the RNA used in the panel can be viewed on OriGene’s Website.

For this study, the real-time PCR was carried out with the ABI Prism 7300 Real-Time PCR (Applied Biosystems/Life Technologies, Saint-Aubain, France) using the Power SYBR Green PCR Master Mix (Applied Biosystems/Life Technologies, Saint-Aubain, France). The cycling conditions were as follow according to the manufacturer’s instruction: 50 °C for 2 min for the activation, 95 °C for 5 min for the pre-soak followed by 42 cycles of 95 °C for 15 s, 60 °C for 1 min and 72 °C for 30 s. As the LightCycler 480 II System, a melting temperature analysis of the amplified gene products was performed at the end of the amplification. The relative expression of the genes of interest normalized to β-Actin (provided by OriGene, Rockville, USA), was determined by the delta Ct method.

### Cytotoxicity assay (LDH assay)

#### IL-17 induced apoptosis assay

MCF7, T47D (1000 cells/well), and MDA-MB468 (3000 cells/well) were seeded in a 96 wells plate in adequate complete medium for 24 h. Then, medium was changed to a FCS-free one alone or treated with recombinant cytokines (20,100 or 500 ng/ml) for 24 h. Untreated cells (control medium), Docetaxel (10 μg/ml) and 1% Triton × 100 treated cells (100% cell death) were used as controls. The cytotoxicity was determined using the Cytotoxicity Detection Kit (Roche, Meylan, France) according to the manufacturer’s instructions. To this aim, 50 μl of supernatant from each well were collected into a 96 wells plate and incubated with 50 μl of freshly prepared Reaction Mixture for 30 minutes at room temperature. Optical density was then read at 490 nm. The percentage of cytotoxicity is calculated as followed: % = 100 × (exp value - control medium value)/(Triton × 100 treated cells value - control medium value).

#### Docetaxel induced cell death

MCF7, T47D, BT20 and IJG-1731 cells (1000 cells/well) were seeded in a 96 wells plate in adequate complete medium alone or treated with recombinant cytokines (1 or 10 ng/ml). After 48 h of culture, medium was changed to a FCS-free one supplemented with corresponding concentration of cytokines. When needed, the U0126 MEK inhibitor was added at 10 μM 24 h before adding the drug. After 24 h, culture medium is then further supplemented with Docetaxel at 5, 10, 20 or 40 μg/ml as indicated. Untreated cells (control medium) and Triton × 100 treated cells (100% cell death) were used as controls. Each condition was performed in duplicates. The cytotoxicity was determined using the Cytotoxicity Detection Kit (Roche, Meylan, France) according to the manufacturer’s instructions as described above.

## Additional Information

**How to cite this article**: Mombelli, S. *et al.* IL-17A and its homologs IL-25/IL-17E recruit the c-RAF/S6 kinase pathway and the generation of pro-oncogenic LMW-E in breast cancer cells. *Sci. Rep.* **5**, 11874; doi: 10.1038/srep11874 (2015).

## Supplementary Material

Supplementary Information

## Figures and Tables

**Table 1:** Primer sequence and product length.

**Gene and GenBank accession**	**Primer sequence**	**Productlength**
IL17-A # NM_002190.2	5'- ACTACAACCGATCCACCTCAC-3'	83 bp
	5'-ACTTTGCCTCCCAGATCACAG-3'	
IL-17E # NM_022789.3	5'-TCCCCCTGGAGATATGAGTTGGACA-3'	175 bp
	5'-GGCATGGCCGCCGGTAGAAG-3'	
IL17RA # NM_014339.5	5'-TGCCCCTGTGGGTGTACTGGT-3'	151 bp
	5'-GCAGGCAGGCCATCGGTGTA-3'	
IL17RB # NM_018725.3	5'-TACCCCGAGAGCCGACCGTT-3'	182 bp
	5'-GGCATCTGCCCGGAGTACCCA-3'	
IL17RC # NM_153460.3	5'-GGCTTGGTTTCACGCGCAGC-3'	192 bp
	5'-CGGCCCTGCAAGAAGTCGGG-3'	
GAPDH	5'-GAAGGTGAAGGTCGGAGTCA-3'	199 bp
	5'-GACAAGCTTCCCGTTCTCAG-3'	
Β-Actin	5'-CAGCCATGTACGTTGCTATCCAGG-3'	151 bp
	5'-AGGTCCAGACGCAGGATGGCATG-3'	
