# Supplementary material for: IL-17A and its homologs IL-25/IL-17E recruit the c-RAF/S6 kinase pathway and the generation of pro-oncogenic LMW-E in breast cancer cells
Source: Sci Rep. 2015 Jul 8;5:11874. doi: 10.1038/srep11874 (PMC4648389; doi:10.1038/srep11874)
Supplement: Supplementary Information [file srep11874-s1.pdf]

IL-17A and its homologs IL-25/IL-17E recruit the c-RAF/S6 kinase pathway and the generation of pro-oncogenic LMW-E in breast cancer cells

Sarah Mombelli[1,2,3,4], Stéphanie Cochaud[1,2,5], Yacine Merrouche[3,4], Christian Garbar[3], Frank Antonicelli[4], Emilie Laprevotte[5,6] Gilles Alberici[5], Nathalie Bonnefoy[6], Jean-François Eliaou[6,7], Jérémy Bastid[5], Armand Bensussan[1,2#*], Jérôme Giustiniani [3,4#].

[1]Institut National de la Santé et de la Recherche Médicale (INSERM) U976, Hôpital Saint Louis, 75010 Paris, France

[2] Université Paris Diderot, Sorbonne Paris Cité, Laboratoire Immunologie Dermatologie & Oncologie, UMR-S 976, F-75475, Paris, France

[3]Institut Jean Godinot, Unicancer, F- 51726 Reims, France

[4]Université Reims-Champagne-Ardenne, DERM-I-C, EA7319, 51 rue Cognacq-Jay, 51095 Reims Cedex, France

[5]OREGA Biotech, F-69130 Ecully, France

[6]IRCM, Institut de Recherche en Cancérologie de Montpellier ; INSERM, U896 ; Université Montpellier 1 ; CRLC Val d'Aurelle Paul Lamarque, Montpellier, F-34298, France.

[7]Département d'Immunologie, Centre Hospitalier Régional Universitaire de Montpellier et Faculté de Médecine Université Montpellier 1, Montpellier, France.

[#]Contributed equally

[*]Corresponding author: armand.bensussan@inserm.fr

Conflict of interest: G.A., N.B., J-F.E. and A.B are cofounders and shareholders of OREGA Biotech. JB is employee of OREGA Biotech and S.M., S.C., Y.M., C.G., F.A., E.L. and J.G. declare no conflict of interest

Supp Figure 1: IL-17E-induced resistance to Docetaxel is dependent of ERK1/2 activation. BT20 cells were stimulated with recombinant IL-17E at 10ng/ml alone or in association with the MEK inhibitor U0126 at 10μM 24h before adding the drug. Cell death is measured after 7h of culture. Data shown are representative of 2 independent experiments.

Supp Figure 2: IL-17E induces the phosphorylation of ERK1/2 and cell proliferation (**A**) Western blot analysis of pThr202/pTyr204 Erk1/2 was performed on MDA-MB468 stimulated for 20 min with recombinant IL-17A or IL-17E at 10ng/ml alone. (**B**) Western blot analysis of pErk1/2 was performed on BT20 cells stimulated for 30 min with recombinant IL-17E at 10ng/ml alone or in association with the MEK inhibitor U0126 at 10μM 24h before adding the drug. The figure shows one representative experiment from two independent experiments.

(**C**) MCF7, T47D, and IJG-1731 breast cancer cell lines were cultured in complete medium supplemented with 20ng/ml of recombinant human IL-17A or IL-17E. Cell proliferation was assessed at 72h using tritiated thymidine ([3H]-TdR) incorporation protocol. Data are the mean ± SEM of two independent experiments, each performed in hexaplicates (* P<0.05).

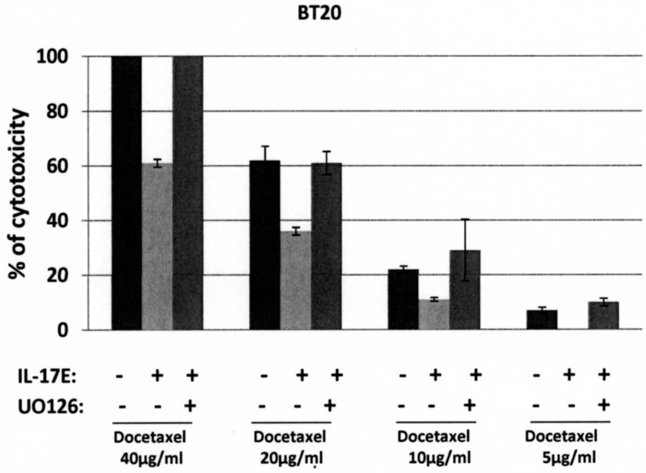

Supp Fig1: IL-17E-induced resistance to Docetaxel is dependent of ERK1/2 activation

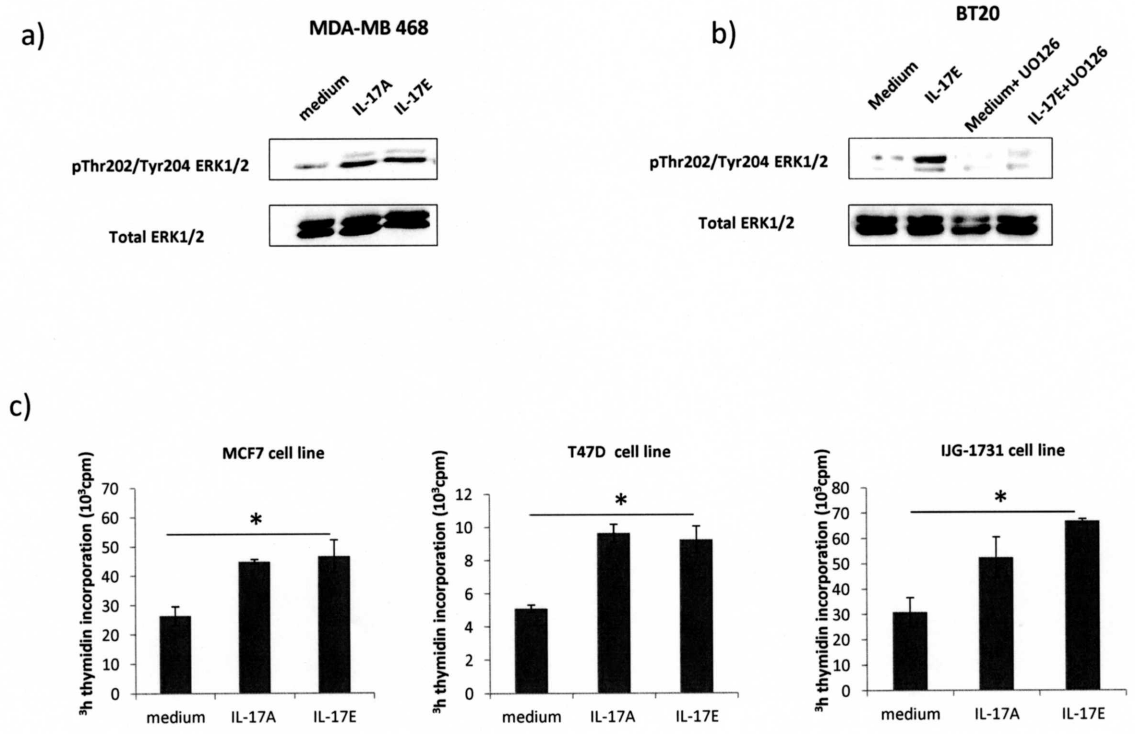

Supp Fig2: IL-17E induces the phosphorylation of ERK1/2 and cell proliferation
